# Biosensor-guided discovery of peptide inhibitors targeting the ribosomal protein uS5-PDCD2 chaperone interaction

**DOI:** 10.1016/j.jbc.2026.111415

**Published:** 2026-03-31

**Authors:** Zabih Mir Hassani, Frédérique Goulet, Duc Tai Nguyen, Anne-Marie Landry-Voyer, Lauren Kwiatek, Shany Gaudet, Pierre-Luc Boudreault, Taha Azad, François Bachand

**Affiliations:** 1 Department of Biochemistry & Functional Genomics, Faculty of Medicine and Health Sciences, Université de Sherbrooke Cancer Research Institute, Université de Sherbrooke, Sherbrooke, Québec, Canada; 2 Department of Pharmacology-Physiology, Faculty of Medicine and Health Sciences, Institut de Pharmacologie de Sherbrooke (IPS), Université de Sherbrooke, Sherbrooke, Québec, Canada; 3 Department of Microbiology and Infectiology, Faculty of Medicine and Health Sciences, Université de Sherbrooke Cancer Research Institute, Université de Sherbrooke, Sherbrooke, Québec, Canada

**Keywords:** programmed cell death 2 (PDCD2), ribosomal protein uS5 (RPS2), dedicated ribosomal protein chaperones, Protein–protein interaction interface, peptide inhibitors, bioluminescence complementation assay, structural modeling

## Abstract

Programmed cell death 2 (PDCD2) is an evolutionarily conserved protein essential for cell viability from yeast to humans. PDCD2 functions as a dedicated chaperone to the 40S ribosomal protein uS5, and loss of PDCD2 function impairs ribosome biogenesis. As cancer cells require a substantial supply of ribosomes to maintain elevated levels of protein synthesis, targeting the protein interaction interface between PDCD2 and uS5 is a promising modality to mitigate ribosome biogenesis. In this study, we used affinity purification assays and structural modeling to identify a stretch of 30 amino acids in the N-terminal region of uS5 that is necessary and sufficient for interaction with PDCD2. Our data also identified a conserved FxxGFG motif in uS5 that is important for association with PDCD2 *via* hydrophobic interactions. Notably, we developed a sensitive complementation-based biosensor that can monitor PDCD2-uS5 interaction in cell extracts and living human cells. Using this biosensor, we identified an 11-amino acid uS5-derived peptide that inhibits the PDCD2–uS5 interaction and impairs cancer cell viability. Such peptides provide a starting point in the development of peptidomimetic inhibitors capable of modulating ribosome biogenesis *via* disruption of ribosomal protein-dedicated chaperone complexes.

Carcinogenesis begins with a series of genetic and epigenetic alterations that disrupt cellular regulatory mechanisms, leading to uncontrolled proliferation and evasion of cell death (1). This multistep process involves increased production of proteins essential for cell cycle progression, metabolism, and survival. Accordingly, cancer cells often upregulate ribosome biogenesis (RiBi) and components of the translational machinery to enhance protein synthesis capacity, which not only supports tumor growth, but also contributes to the malignant phenotype (2, 3, 4). The augmented ribosome production in cancer cells is evidenced by increased RNA polymerase I (RNAPI) activity (5, 6), overexpression of ribosomal protein (RP) genes (2, 4), and irregular nucleolar morphology (7). RiBi is therefore increasingly recognized as an emerging strategy for anti-cancer treatment (8, 9), as exemplified by the development of RNAPI inhibitors CX-5461 and BMH-21, which are currently being evaluated in preclinical trials (10, 11).

Structurally, the actively translating eukaryotic ribosome is composed of a small (40S) and a large (60S) subunit. In mammals, the small subunit consists of the 18S ribosomal RNA (rRNA) and 33 ribosomal proteins (RPs), whereas the large subunit contains three different rRNAs (5S, 5.8S and 28S rRNAs) and 47 RPs (12). The assembly of mammalian ribosomes begins in the nucleolus where RNAPI transcribes multiple copies of rDNA genes into primary 47S pre-rRNAs (13). These primary pre-rRNA transcripts are subjected to co-transcriptional processing, including base modifications and ribonuclease cleavage by a large ensemble of assembly factors (12). Following endonucleolytic cleavage of the 47S precursor rRNA between the 18S and 5.8S rRNA sequences, 40S and 60S pre-particles subsequently follow distinct maturation pathways, which are coordinated with the stepwise incorporation of subunit-specific RPs (14). Thus, considering that RiBi involves the coordinated action of all three RNA polymerases, hundreds of ribosome maturation factors, over 200 small nucleolar RNAs (snoRNAs), and the production of 80 different RPs, ribosome synthesis is considered one of the most energy-demanding and complex processes occurring in eukaryotic cells (15).

The expression and subcellular trafficking of RPs is a particularly challenging aspect of eukaryotic RiBi. Specifically, the 80 different RP genes are independently transcribed in the nucleus by RNAPII, RP mRNAs are then exported to the cytosol for translation into newly synthesized free RPs that need to be re-imported into the nucleus for incorporation into ribosomal subunit precursors in a highly orchestrated and sequential manner (14). As RPs are among the most highly expressed cellular proteins, they can potentially be involved in non-specific interactions and aggregation due to their generally basic nature, disordered regions, and flexible terminal extensions (16). Eukaryotic cells have therefore developed mechanisms to secure a sustainable supply of RPs for ribosome production, including general and dedicated chaperones (17).

In the past few years, dedicated RP chaperones have emerged as a class of conserved proteins fundamental to RiBi, with each dedicated chaperone typically binding a single or a small subset of RPs, shielding critical domains until proper incorporation into pre-ribosomal particles (17). Studies in yeast have identified several dedicated chaperones, including Acl4, Syo1, Tsr2, and Tsr4, each with defined roles in escorting specific RPs to the nucleus (18, 19, 20, 21, 22). In humans, however, orthologs of these factors are less well-characterized, but emerging evidence suggest that dedicated chaperones are evolutionarily conserved (23). Notably, we have shown that the Programmed Cell Death 2 (PDCD2) protein, which is the ortholog of *Saccharomyces cerevisiae* Tsr4, functions as a dedicated chaperone to human uS5 (RPS2) (24). Specifically, biochemical data support a model whereby PDCD2 is recruited to nascent uS5 co-translationally, stabilizes the soluble pool of free uS5, and promotes its incorporation into 40S ribosomal subunit (24). Accordingly, loss of PDCD2 expression leads to defects in the synthesis of the small ribosomal subunit that phenocopy a uS5 deficiency (24, 25), suggesting that PDCD2 and uS5 contribute to RiBi *via* the same mechanism. Most dedicated RP chaperones are encoded by essential genes in *S. cerevisiae* (18, 19, 20, 21, 22), reflecting the critical role of these proteins in RiBi. Consistent with this idea, *PDCD2* is essential for stem cell viability and proliferation, and its absence leads to early embryonic lethality in mice (26). In humans, bi-allelic variants in *PDCD2* that impair the association of PDCD2 with uS5 are associated with embryonic defects and recurrent pregnancy loss (27). Furthermore, according to the Dependency Map (DepMap) project (28), CRISPR-Cas9 loss-of-function screens indicate that *PDCD2* is broadly required for the survival of many human cancer cell lines, underscoring the potential of targeting the PDCD2–uS5 interaction as an anti-cancer therapeutic strategy. However, the molecular determinants of uS5 that promote the stable association with PDCD2 remains unclear.

In this study, we combined the use of biochemical assays and protein structural predictions to disclose a 30-amino acid region in human uS5 that is necessary and sufficient for recognition by PDCD2. This region is in the eukaryotic-specific N-terminal extension of uS5 and includes a conserved FxxGFG motif. The aromatic rings of the pair of phenylalanine residues in the FxxGFG motif are buried into pocket-like hydrophobic cavities of PDCD2. We show that substitutions of these conserved phenylalanine residues in uS5 significantly perturb the association with PDCD2. We also developed a robust bioluminescence-based biosensor derived from the Nanoluciferase binary technology (NanoBiT) to monitor the uS5-PDCD2 interaction. Using this biosensor, we identified short uS5-derived peptides that can efficiently inhibit the uS5-PDCD2 interaction and reduce the viability of cancer cells. Our findings define the molecular features that underlie uS5 recognition by PDCD2 and highlight the critical contribution of the FxxGFG motif to this dedicated chaperone-ribosomal protein interaction. At the same time, the NanoBiT-based biosensor we developed provides a powerful platform to monitor and perturb this interaction in a high-throughput format, thereby enabling both mechanistic dissection and discovery of potential modulators of ribosome assembly.

## Results

### Residues 21 to 50 of uS5 are necessary and sufficient for interaction with human PDCD2

**Figure 1:**
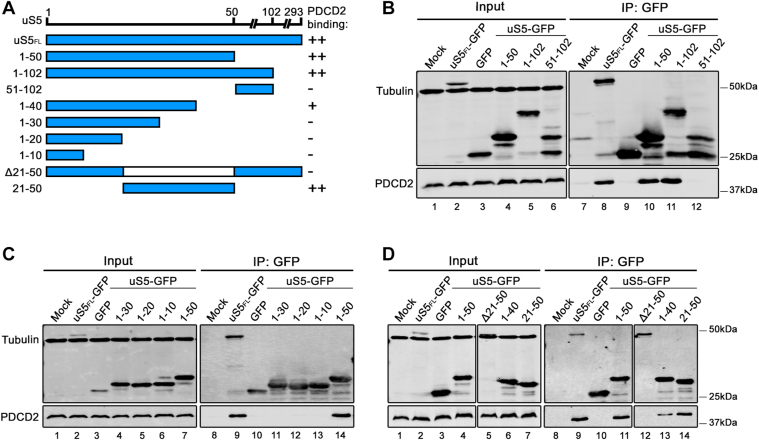
**Amino acids 21 to 50 of uS5 are necessary and sufficient for binding human PDCD2.***A*, summary of uS5 variants analyzed in binding assays and extent of interactions with human PDCD2. Schematic representation of full-length human uS5 containing 293 residues. Amino- and carboxy-terminal deletions in uS5 are indicated along with PDCD2 binding abilities these variants demonstrated after immunoprecipitations assays from human cell extracts. All these proteins were engineered to express GFP at the C-terminal end of uS5. *B*, Western blot analysis of total extracts (lanes 1–6) and anti-GFP precipitates (lanes 7–12) prepared from HEK293T cells that were transiently transfected for 48 h with the indicated versions of GFP-tagged uS5 constructs or GFP alone (lanes 3 and 9). Mock (lanes 1 and 7) refers to non-transfected HEK293T cells. The blot was analyzed for Tubulin and GFP (*top*) and endogenous PDCD2 (*bottom*). *C*, Western blot analysis of total extracts (lanes 1–7) and anti-GFP precipitates (lanes 8–14) prepared from HEK293T cells that were transiently transfected for 48 h with the indicated versions of GFP-tagged uS5 constructs or GFP alone (lanes 3 and 10). Mock (lanes 1 and 8) refers to non-transfected HEK293T cells. The blot was analyzed for Tubulin and GFP (*top*) and endogenous PDCD2 (*bottom*). *D*, Western blot analysis of total extracts (lanes 1–7) and anti-GFP precipitates (lanes 8–14) prepared from HEK293T cells that were transiently transfected for 48 h with the indicated versions of GFP-tagged uS5 constructs or GFP alone (lanes 3 and 10). Mock (lanes 1 and 8) refers to non-transfected HEK293T cells. The blot was analyzed for Tubulin and GFP (*top*) and endogenous PDCD2 (*bottom*).

We previously used computational modeling based on AlphaFold Multimer (29) to predict the structure of a PDCD2–uS5 complex (23), which suggested that the N-terminal region of uS5 is important for recognition by PDCD2. This prediction is consistent with biochemical data indicating that the first N-terminal 100-amino acids of *S. cerevisiae* uS5 are required for interaction with the yeast PDCD2 homolog, Tsr4 (18, 21). To test the predicted model of the human PDCD2-uS5 complex (23) directly, we used coimmunoprecipitation assays by employing a set of plasmid constructs expressing different domains of uS5 that were C-terminally tagged with GFP (Fig. 1*A*). Based on data of the yeast uS5-Tsr4 complex and our AlphaFold Multimer prediction of human PDCD2-uS5, we designed constructs that included amino acids 1 to 50, 1 to 102, and 51 to 102 (Fig. 1*A*). Plasmid constructs were transfected into HEK293T cells, and total extracts were subjected to anti-GFP purification, which were analyzed for copurification of endogenous PDCD2 by immunoblotting. As controls, endogenous PDCD2 was efficiently copurified with full-length uS5 fused to GFP (Fig. 1*B*, lane 8), whereas GFP alone did not copurify PDCD2 (Fig. 1*B*, lanes 9). As shown in Figure 1*B*, residues 1 to 50 and 1 to 102 of uS5 copurified endogenous PDCD2 at similar levels to full-length uS5 (compare lanes 10–11 to lane 8). In contrast, residues 51 to 102 of uS5 could not form a stable complex with PDCD2 (Fig. 1*B*, lane 12). These data indicate that the first 50-amino acids of uS5 are important for recognition by human PDCD2.

We next performed 10-amino acid truncations using uS5_1-50_, generating constructs that expressed residues 1 to 40, 1 to 30, 1 to 20, and 1 to 10 of uS5 (Fig. 1*A*). As shown in Figure 1*C*, PDCD2 was not detected in affinity purification assays using residues 1 to 30, 1 to 20, and 1 to 10 of uS5 (Fig. 1*C*, lanes 11–13). Although endogenous PDCD2 was copurified with uS5_1-40_, copurification levels of PDCD2 were reduced compared to uS5_1-50_ (Fig. 1*D*, compare lane 13 to lane 11). We thus conclude that, although not critical, residues 40 to 50 of uS5 are important for a robust association with PDCD2.

As the first 30 amino acids of uS5 were not sufficient to promote a stable uS5-PDCD2 interaction (Fig. 1*C*), and given that residues 21 to 50 in the N-terminal disordered region of uS5 exhibited the greatest structural change after association with PDCD2 based on AlphaFold modeling (23), we next deleted residues 21 to 50 in the context of full length uS5 (Fig. 1*A*). As shown in Figure 1*D*, deletion of residues 21 to 50 in uS5 completely abrogated its ability to copurify endogenous PDCD2 (lane 12) compared to full-length uS5 (lane 9). Conversely, we found that fusion of residues 21 to 50 of uS5 to GFP was sufficient to promote a robust uS5-PDCD2 complex (Fig. 1*D*, lane 14). Taken together, our affinity purification assays demonstrate that residues 21 to 50 of uS5 are necessary and sufficient for interaction with human PDCD2.

### A conserved FxxGFG motif in uS5_21-50_ and a hydrophobic core region of PDCD2 are important for the formation of a stable uS5–PDCD2 complex

**Figure 2:**
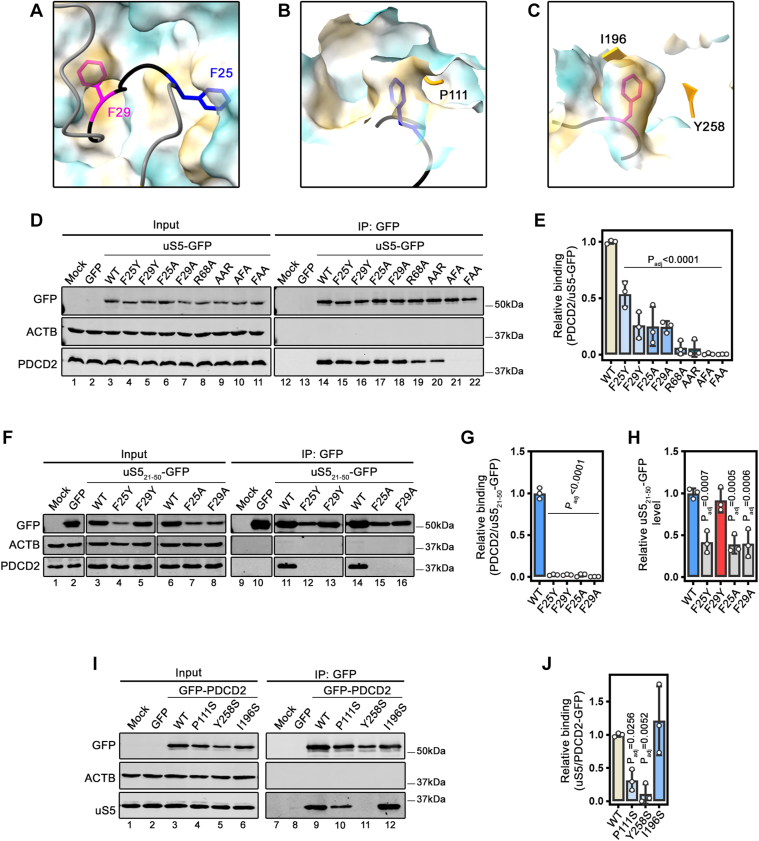
**Interactions between a conserved FxxGFG motif in uS5 and a hydrophobic core region of PDCD2 are important for the formation of a stable uS5-PDCD2 complex.***A*, alphaFold-Multimer prediction of uS5-PDCD2 complex showing the insertion of F25 (*blue*) and F29 (*pink*) of human uS5 into hydrophobic core regions of PDCD2. *B-C*, residues of PDCD2 predicted to participate in hydrophobic interactions with F25 (*B*) and F29 (*C*) of uS5 are shown. *D*, Western blot analysis of total extracts (lanes 1–11) and anti-GFP precipitates (lanes 12–22) prepared from HEK293T cells that were transiently transfected for 48 h with either wild-type uS5 fused to GFP (lanes 3 and 14) or the indicated F25 and F29 uS5 variants. AAR: F25A F29A R68; AFA: F25A F29 R68A; FAA: F25 F29A R68A. Mock (lanes 1 and 12) refers to non-transfected HEK293T cells. The blot was analyzed for GFP (top), beta-actin (*middle*), and PDCD2 (*bottom*). *E*, quantification of PDCD2 levels recovered in GFP immunoprecipitates were normalized to the levels of uS5-GFP. Values were expressed relative to the wild-type (WT) version of uS5, which was set to 1.0. The data and error bars represent the average and SD from three independent experiments. *p*-values are indicated, as determined by a one-way ANOVA with Dunnett’s multiple comparisons test. *F*, Western blot analysis of total extracts (lanes 1–8) and anti-GFP precipitates (lanes 9–16) prepared from HEK293T cells that were transiently transfected for 48 h with wild-type (WT) and the indicated F25 and F29 variants of uS5_21-50_-GFP. Mock (lanes 1 and 9) refers to non-transfected HEK293T cells. The blot was analyzed for GFP (*top*), beta-actin (*middle*), and PDCD2 (*bottom*). Note that the WT input sample shown in lane 3 is the same as that shown in lane 6, and the WT IP sample shown in lane 11 is the same as that shown in lane 14 and were included in multiple panels for comparison. The unprocessed original images are provided in the Supplementary Information. *G*, quantification of PDCD2 levels recovered in GFP immunoprecipitates were normalized to the levels of uS5_21-50_-GFP. Values were expressed relative to the wild-type (WT) version of uS5_21-50_, which was set to 1.0. The data and error bars represent the average and SD from three independent experiments. *p*-values are indicated, as determined by a one-way ANOVA with Dunnett’s multiple comparisons test. *H*, cellular levels of wild-type and indicated F25/F29 variants of uS5 expressed in the context of uS5_21-50_-GFP, normalized to beta-actin (ACTB). Values were expressed relative to the wild-type (WT) version of uS5_21-50_, which was set to 1.0. The data and error bars represent the average and SD from three independent experiments. *p*-values are indicated, as determined by a one-way ANOVA with Dunnett’s multiple comparisons test. *I*, Western blot analysis of total extracts (lanes 1–6) and anti-GFP precipitates (lanes 7–12) prepared from HEK293T cells that were transiently transfected for 48 h with either wild-type or the indicated variants of PDCD2. Mock (lanes 1 and 7) refers to non-transfected HEK293T cells. The blot was analyzed for GFP (*top*), beta-actin (*middle*), and uS5 (*bottom*). *J*, quantification of uS5 levels recovered in GFP immunoprecipitates were normalized to the levels of GFP-PDCD2. Values were expressed relative to the wild-type (WT) version of PDCD2, which was set to 1.0. The data and error bars represent the average and SD from three independent experiments. *p*-values are indicated, as determined by a one-way ANOVA with Dunnett’s multiple comparisons test.

We previously noticed a conserved FxxGFG motif in the eukaryotic-specific N-terminal extension of uS5 in multicellular organisms (see Fig. S1) that is a more basic GFG motif in budding and fission yeasts (23). Interestingly, the two phenylalanine residues (F25 and F29) in the FxxGFG motif of human uS5 appear to be buried inside pocket-like hydrophobic core regions of PDCD2 (Fig. 2, *A*–*C*). Amino acids contact analysis using the predicted uS5-PDCD2 model revealed that F25 and F29, along with R38 and R68, are among the most active uS5 residues in terms of proximity to PDCD2, as determined by the number of predicted contacts in a distance range below 4.0 Å (Fig. S2*A*). Specifically, both F25 and F29 of uS5 are enclosed within a pair of hydrophobic layers, resulting in a “hydrophobic sandwich” configuration (Fig. S2*B*), while R38 and R68 of uS5 participate in salt bridges that may play a key role in molecular recognition by PDCD2 (Fig. S2*C*). To begin to assess the functional significance of uS5 F25 and F29 residues for PDCD2 association, we generated a set of uS5 variants in which F25 and/or F29 were substituted to either alanine or tyrosine residues. Copurification assays showed decreased levels of endogenous PDCD2 in uS5 precipitates when F25 or F29 were substituted (Fig. 2*D*, lanes 15–18; quantifications in Fig. 2*E*). Interestingly, the uS5 AAR variant, in which F25 and F29 were simultaneously substituted to alanine residues, demonstrated a greater decrease in PDCD2 enrichment compared to F25A and F29A single mutants (Fig. 2*D*, compare lane 20 to lanes 17–18; Fig. 2*E*), suggesting an additive role for those conserved phenylalanine residues in PDCD2 interaction. As predicted by the amino acid contact analysis (Fig. S2), we also found that uS5 R68 significantly contributes to the formation of a stable uS5–PDCD2 complex (Fig. 2*D*, see lane 19; Fig. 2*E*). Strikingly, when uS5 R68 was substituted in combination with either F25 or F29 (AFA or FAA variants, respectively), the association between uS5 and PDCD2 was completely abrogated (Fig. 2*D*, lanes 21–22; Fig. 2*E*). We also analyzed a set of F25 and F29 single mutants in the context of the uS5 minimal domain (uS5_21-50_). In this setting, single substitutions of F25 and F29 to either tyrosine or alanine resulted in a complete loss of interaction with endogenous PDCD2 (Fig. 2*F*, see lanes 12–13 and 15–16; quantifications in Fig. 2*G*). In fact, except for F29Y, the F25Y, F25A, and F29A variants all showed reduced protein levels in the context of the uS5 minimal domain (Fig. 2*F*, see lanes 3–8; Fig. 2*H*), suggesting decreased protein stability for those variants.

Additionally, we generated a set of PDCD2 variants in amino acids predicted to shape the hydrophobic pockets in which the aromatic side chain of F25 and F29 of uS5 are inserted into (Fig. 2, *B* and *C* and Fig. S2*B*). We targeted Proline-111 (P111), Isoleucine-196 (I196), and Tyrosine-258 (Y258), which were substituted to serine, a smaller polar residue. PDCD2 constructs were transfected in HEK293T cells and total extracts were subjected to anti-GFP purification, which were analyzed for copurification of endogenous uS5 by immunoblotting. As shown in Figure 2*I*, the PDCD2 Y258S variant was defective at interacting with uS5 (lane 11; Fig. 2*J*), while the P111S variant of PDCD2 showed a 3-fold reduction in relative uS5 binding (compare lanes 9–10; Fig. 2*J*). In contrast, the PDCD2 I196S variant did not significantly affect formation of a uS5-PDCD2 complex (Fig. 2*I*, lane 12; Fig. 2*J*). Collectively, the data in Figure 2 demonstrate that the phenylalanine residues within the conserved FxxGFG motif of uS5 are critical for forming a stable uS5-PDCD2 complex, through interactions with residues that shape the hydrophobic pockets of PDCD2.

### Excess dosage of uS5_21-50_ inhibits the formation of the uS5-PDCD2 complex and affects PDCD2 subcellular localization

**Figure 3:**
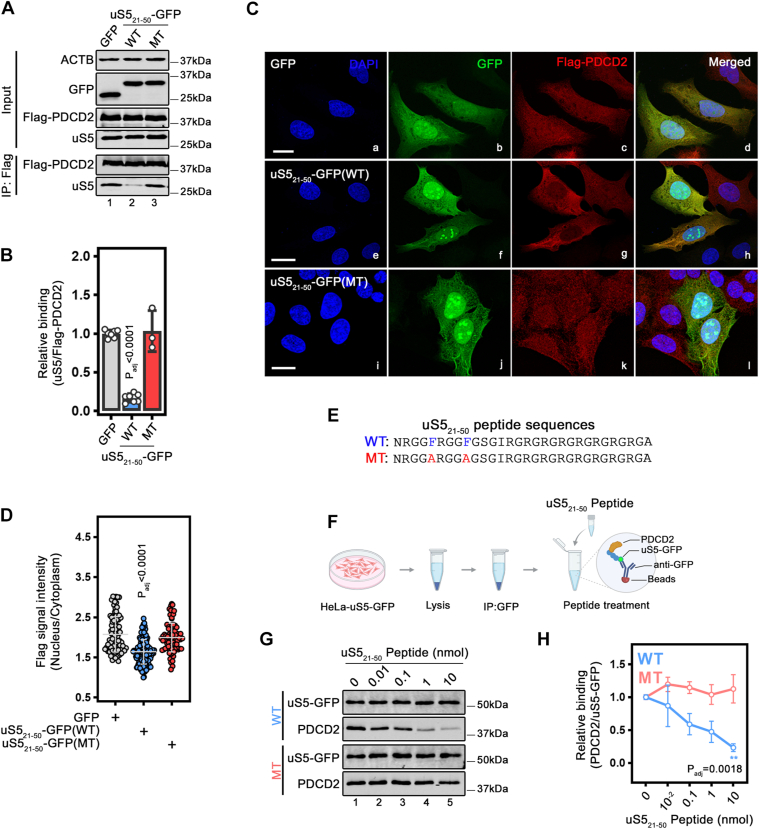
**Inhibition of the uS5-PDCD2 interaction using residues 21 to 50 of uS5.***A*, Western blot analysis of total extracts (Input, *Top*) and anti-Flag purifications (IP:Flag, *Bottom*) prepared from HeLa cells that stably expressed Flag-PDCD2 and that were previously transfected with constructs encoding either GFP alone (lane 1), wild-type (WT, lane 2) or F29Y (MT, lane 3) versions of uS5_21-50_-GFP. The blots were analyzed for GFP, Actin, uS5, and Flag-PDCD2. *B*, quantification of relative uS5 levels copurified from anti-Flag precipitates normalized to Flag-PDCD2 levels. Values were expressed relative to the GFP control vector, which was set to 1.0. The data and error bars represent the average and SD from at least three independent experiments. *p*-value is indicated, as determined by a one-way ANOVA with Dunnett’s multiple comparisons test. (*C*) U2OS cells that conditionally express Flag-PDCD2 were transfected with either GFP control vector (panels a-d), wild-type (panels e-h) or F29Y (panels i-l) versions of uS5_21-50_-GFP. At the time of transfection, doxycycline was added to the media to induce the expression of Flag-PDCD2. 48 h post-transfection, cells were fixed and simultaneously analysed by direct fluorescence (b, f and j) and immunostaining for Flag-PDCD2 (c, g and k). DNA staining with DAPI shows the nucleus of each cell (a, e and i). Scale bars correspond to 20 μm. *D*, quantification of nucleus-to-cytoplasmic ratios of Flag-PDCD2 shows a significant decrease in cells that expressed the wild-type version of uS5_21-50_-GFP. More than 60 cells were analyzed for each condition from three independent immunofluorescence experiments. Statistical differences were calculated using a one-way ANOVA with Dunnett’s multiple comparisons test. *p*-value is indicated. *E*, Peptide sequences corresponding to residues 21 to 50 of uS5, showing conserved phenylalanines in blue (WT) and substitutions to alanine residues in the mutant in red (MT). *F*, schematic of the peptide competition experiments using purified uS5-GFP/PDCD2 complex. See text for details. *G*, Western blot analysis of an uS5-GFP immunoprecipitate that were washed, divided, and treated with increasing concentrations of either wild-type (*Top*, WT) or mutant (*Bottom*, MT) uS5_21-50_ peptides (lanes 2–5) or with no peptide (lane 1). Blots were analyzed for uS5-GFP and endogenous PDCD2. (*H*) Quantification of PDCD2 levels copurified from anti-GFP precipitates normalized to uS5-GFP levels. The values were then set to 1.0 for the control purification in the absence of peptide. Solid lines mark the average binding from three independent replicates, with error bars corresponding to standard deviations. Statistical differences were calculated using a one-way ANOVA with Dunnett’s multiple comparisons test. *p*-value is indicated.

Having identified residues 21 to 50 of uS5 (uS5_21-50_) as both necessary and sufficient for interaction with PDCD2 (Figs. 1 and 2), we examined the potential of uS5_21-50_ to compete with the PDCD2-uS5 interaction. To test this, we overexpressed uS5_21-50_-GFP in human cells that stably express a Flag-tagged version of PDCD2 and analyzed the levels of endogenous uS5 copurified with Flag-PDCD2. As shown in Figure 3*A*, excess dosage of the uS5 minimal domain significantly decreased the levels of endogenous uS5 copurified with Flag-PDCD2 compared to cells that overexpressed GFP alone (compare lanes 1–2; quantification in Fig. 3*B*). To assess the specificity of this competition, we also overexpressed a version of uS5_21-50_-GFP with the F29Y substitution, which did not bind PDCD2 but showed expression comparable to wild-type uS5_21-50_-GFP (Fig. 2, *F*–*H*). As shown in Figure 3*A*, excess dosage of the F29Y version of uS5_21-50_-GFP did not compete the formation of the PDCD2-uS5 complex (lane 3; quantifications in Fig. 3*B*). These results suggest that residues 21 to 50 of uS5 can function as a tool to sequester PDCD2 and target the formation of PDCD2–uS5 complex.

To further characterize the effects of excess dosage of uS5_21-50_-GFP on PDCD2 activity, we analyzed the subcellular localization of Flag-PDCD2. For the immunofluorescence analysis of PDCD2, we used a U2OS stable cell line expressing Flag-PDCD2 because their large nucleus and flat morphology facilitate imaging and quantification. 24 h post-transfection of constructs expressing either GFP alone, wild-type or mutant versions of uS5_21-50_-GFP, cells were fixed, permeabilized, and analyzed by direct fluorescence for GFP (Fig. 3*C*, panels b, f, and j) and by anti-Flag immunostaining (Fig. 3*C*, panels c, g, and k). Indirect immunofluorescence of Flag-PDCD2 in U2OS cells produced both nuclear and cytoplasmic signal (Fig. 3*C*, panel c), consistent with previous observations (24). Notably, cells transfected with the wild-type version of uS5_21-50_-GFP showed reduced nuclear staining of Flag-PDCD2, whereas adjacent non-transfected cells did not show such reduction in nuclear Flag-PDCD2 staining (Fig. 3*C*, panels e-h). In contrast, cells transfected with the F29Y mutant version of uS5_21-50_-GFP showed normal nuclear and cytoplasmic Flag-PDCD2 signal (Fig. 3*C*, panels i-l), similar to cells transfected with GFP alone (Fig. 3*C*, panels a-d). Quantification of nuclear and cytoplasmic Flag-PDCD2 signal in GFP-stained cells from independent experiments revealed a significant reduction in the nuclear-to-cytoplasmic ratio of PDCD2 when cells expressed the wild-type version of uS5_21-50_-GFP (Fig. 3*D*). We also noticed that a fraction of uS5_21-50_-GFP localized to subnuclear regions reminiscent of nucleolar staining (Fig. 3*C*, panel f). To assess whether this concentrated uS5_21-50_-GFP nuclear signal corresponded to nucleoli, the uS5_21-50_-GFP fluorescence was combined with an immunostaining for the nuclear marker Fibrillarin. Comparison of the different fluorescent signals showed that uS5_21-50_-GFP was concentrated in nuclear regions that colocalized with anti-Fibrillarin staining (Fig. S3, panels e-h), whereas the targeting of GFP alone to the nucleolus was less specific (Fig. S3, panels a-d). Interestingly, we found that the F29Y version of uS5_21-50_-GFP that does not interact with PDCD2 (Fig. 2) is also targeted to nucleoli (Fig. S3, panels i-l), suggesting that PDCD2 is not required for nucleolar targeting of uS5_21-50_-GFP.

Next, we examined whether we could disrupt pre-formed uS5-PDCD2 complexes using the minimal PDCD2-interacting region of uS5 (residues 21–50). We therefore generated synthetic peptides corresponding to either wild-type (WT) or F25A/F29A double mutant (MT) versions of residues 21 to 50 of uS5 (see Fig. 3*E*) and tested the ability of these peptides to disrupt PDCD2-uS5 interaction *in vitro*. For this, we affinity purified uS5-GFP from human cell extracts and subjected bead-associated PDCD2/uS5-GFP complexes to increasing concentrations of either WT or MT peptides (see Fig. 3*F*). Beads were then washed to remove unbound material and analyzed for PDCD2/uS5-GFP complex formation by immunoblotting. As shown in Figure 3*G*, we found that the wild-type uS5-derived peptide disrupted the uS5–PDCD2 complex in a dose-dependent manner (WT, lanes 1–5; quantification in Fig. 3*H*). In contrast, the F25A/F29A double mutant (MT) version of the uS5-derived peptide did not disrupt the uS5-PDCD2 interaction (Fig. 3, *G* and *H*). Taken together, the data shown in Figure 3 indicate that uS5_21-50_ can inhibit the formation of the uS5-PDCD2 complex and alter the subcellular localization of PDCD2.

### A complementation-based biosensor allows the identification of uS5-derived peptides inhibiting the PDCD2-uS5 interaction

**Figure 4:**
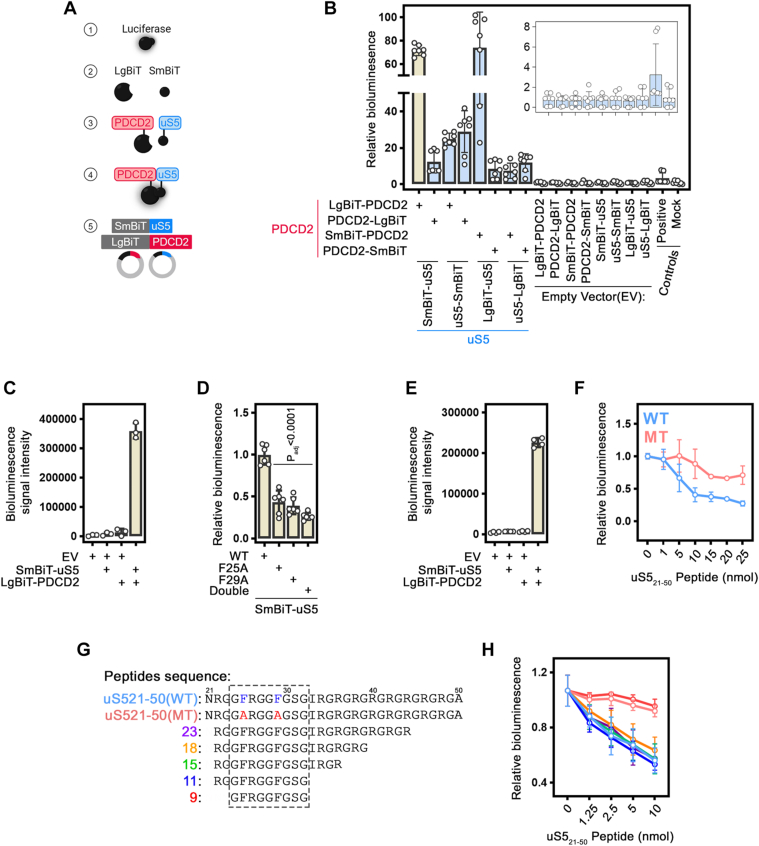
**A complementation-based NanoBiT biosensor to analyse uS5-PDCD2 interaction.***A*, schematic representation of the uS5-PDCD2 NanoBiT-based biosensor. The NanoBiT luciferase (1) is split into two complementary luciferase fragments, LgBiT and SmBiT (2), which are fused to proteins of interest (3), uS5 and PDCD2. Upon interaction between the two proteins (4), the fragments reassemble to form an active NanoLuc luciferase, generating bioluminescence in the presence of the substrate. *B*, relative bioluminescence signals measured in extracts from HEK293T cells co-transfected for 48 h with the indicated NanoBiT-based constructs. Luminescence values were normalized to the mock-transfected control. The PD-1/PD-L1 interaction (48) was included as a positive control. Inset highlights the control results. Data represent the mean ± SD from at least five independent experiments. *C*, bioluminescence signal intensity measured from living human HEK293T cells that were previously cotransfected with the indicated plasmid constructs for 48 h. The data and error bars represent the average and SD from at least three independent experiments. EV, Empty Vector. *D*, relative bioluminescence using extracts of HEK293T cells that were previously transfected with LgBiT-PDCD2 and the indicated wild-type (WT) and mutant versions of SmBiT-us5. The data and error bars represent the average and SD from at least five independent experiments. Statistical differences were calculated using a one-way ANOVA with Dunnett’s multiple comparisons test. *p*-values are indicated. *E*, bioluminescence signal intensity measured by mixing extracts of cells that were independently transfected with the indicated plasmid constructs for 48 h. The data and error bars represent the average and SD from at least four independent experiments. (*F*) Relative bioluminescence measured after mixing cell extracts containing SmBiT-uS5 with extracts containing LgBiT-PDCD2 that were previously incubated with the indicated concentration of the uS5-derived wild-type (*blue curve*) and mutant (*red curve*) peptides. The values were then set to 1.0 for the control reconstitution in the absence of peptide. Solid lines mark the average binding from three independent replicates, with error bars corresponding to standard deviations. *G*, Sequences of peptides truncations from residues 21 to 50 of uS5. The 30 amino acids-long uS5-derived peptides, showing conserved phenylalanines in *blue* (WT) and substitutions to alanine residues in the mutant in *red* (MT), are shown at the *top*. *H*, Relative bioluminescence measured after mixing cell extracts containing SmBiT-uS5 with extracts containing LgBiT-PDCD2 that were previously incubated with the indicated concentration of the uS5-derived wild-type. Colors correspond to peptide lengths shown in *panel G.* The values were then set to 1.0 for the control reconstitution in the absence of peptide. Solid lines mark the average binding from three independent replicates, with error bars corresponding to standard deviations.

Despite establishing a robust biochemical assay based on affinity purification to study the inhibition of the uS5-PDCD2 interaction *in vitro* (Fig. 3, *F*–*H*), this represents a lengthy assay that is not compatible with high-throughput screenings. We therefore developed a protein-fragment complementation assay referred as NanoLuc Binary Technology (NanoBiT) (30), which is based on NanoLuc, an engineered luciferase derived from a deep sea shrimp (31). NanoBiT biosensors rely on protein-protein interaction (PPI)-dependent reconstitution of the NanoLuc enzyme, which upon exposure to a substrate, allows quantitative analysis of PPI by emission of bright luminescence (30). In the NanoBiT reporter system, the NanoLuc enzyme is fragmented into a Large fragment (LgBiT; 18 kDa) and a small complementary peptide (SmBiT; 11 amino acids), which has low affinity for the LgBiT (see Fig. 4*A*, panels 1–2). Yet, when fused to interacting proteins of interest, the subunits come together to reconstitute an active enzyme and generate bright luminescence signal in the presence of substrate (Fig. 4*A*, panels 3–4). To optimize the NanoBiT biosensor for specificity and dynamic range, it is important to find a favorable combination of particles (*e.g.* LgBiT on PDCD2 and SmBiT on uS5) and the optimal orientation for particle fusion (*i.e.* C-terminus vs N-terminus). We therefore designed expression constructs with all 8 possible combinations and orientations, which were co-transfected into HEK293T cells. 48 h post-transfection, cell lysates were incubated with the coelenterazine (CTZ) substrate and analyzed for luminescence signal. As shown in Figure 4*B* (see inset), cell lysates containing a single fragment (LgBiT or SmBiT fusions) only produced background signal. In contrast, all 8 combinations of uS5 and PDCD2 NanoBiT reporters resulted in bioluminescence signal, with two combinations standing out: LgBiT-PDCD2 + SmBiT-uS5 and SmBiT-PDCD2 + LgBiT-uS5 (Fig. 4*B*). Importantly, the difference in NanoLuc reconstitution between the different combinations of LgBiT and SmBiT fusions was not correlated with expression levels (Fig. S4). Because we observed more variability using the SmBiT-PDCD2 + LgBiT-uS5 combination, we opted for the LgBiT-PDCD2 + SmBiT-uS5 for further characterization.

We next tested the performance of our uS5-PDCD2 biosensor in living human cells. For this, HEK293T cells were co-transfected with constructs expressing LgBiT-PDCD2 and SmBiT-uS5. 48 h post-transfection, cells were treated with diluted CTZ for 30 min, washed, and analyzed for bioluminescence reading without cell lysis. Biosensor assays in living HEK293T cells resulted in bioluminescence signal measurements similar to assays using lysates prepared from co-transfected cells (Fig. 4*C*). We also examine the specificity of the uS5-PDCD2 biosensor signal by generating the F25A, F29A, and F25A/F29A double mutants in the SmBiT-uS5 construct, which were co-transfected with the wild-type LgBiT-PDCD2 construct. Consistent with our affinity purification assays (Fig. 2), we found a 2-fold decrease in signal intensity with the F25A and F29A single mutants, and a roughly 4-fold decrease with the F25/F29A double mutant (Fig. 4*D*).

Next, we tested whether a uS5_21-50_ synthetic peptide can inhibit the reconstitution of the uS5-PDCD2 biosensor. To do this, we first tested whether we could reconstitute the uS5-PDCD2 biosensor by mixing extracts prepared from cells that were independently transfected with the LgBiT-PDCD2 and SmBiT-uS5 constructs. As shown in Figure 4*E*, the LgBiT-PDCD2 and SmBiT-uS5 reconstituted a functional NanoLuc enzyme by combining cell extracts that independently expressed the two subunits. As expected, reconstitution of a uS5-PDCD2 biosensor using independent extracts was in a dose-dependent manner (Fig. S5). Given the ability to reconstitute a functional uS5-PDCD2 biosensor using extracts from independently transfected human cells, we next tested whether we could inhibit biosensor reconstitution using the uS5_21-50_ synthetic peptide. For this, we treated extracts of cells previously transfected with the construct expressing LgBiT-PDCD2 with increasing concentrations of either wild-type (WT) or mutant (MT; F25A/F29A) uS5_20-51_ synthetic peptide for 15 min. Equal volumes of lysates prepared from cells that expressed SmBiT-uS5 were then added to each well and incubated for 15 min at room temperature to allow uS5-PDCD2 biosensor formation, which was followed by CTZ incubation and bioluminescence measurements. The wild-type uS5_21-50_ synthetic peptide effectively inhibited reconstitution of the uS5-PDCD2 biosensor at concentrations between 1 to 10 nmol (Fig. 4*F*, blue curve), whereas the mutant peptide showed minimal inhibition at these concentrations (Fig. 4*F*, red curve). At concentrations >10 nmol of wild-type uS5_21-50_ peptide, the inhibition of uS5-PDCD2 biosensor reconstitution appeared to plateau relative to 10 nmol of uS5-derived peptides (Fig. 4*F*, blue curve) and resulted in non-specific inhibition using the mutant peptide (Fig. 4*F*, red curve). We thus conclude that 1 to 10 nmol of synthetic uS5_21-50_ peptide is the optimal range to block the reconstitution of the complex between LgBiT-PDCD2 and SmBiT-uS5, which is consistent with results obtained using biochemical assays (Fig. 3).

Our current synthetic peptides consist of a 30 amino acid-long region corresponding to residues 21 to 50 of uS5. Because longer peptides generally face greater challenges in penetrating human cell membranes compared to shorter ones (32), we used our uS5-PDCD2 biosensor to test shorter versions of uS5-derived peptides. We therefore truncated the uS5_21-50_ peptide at both N- and C-termini to generate synthetic peptides of 23, 18, 15, 11, and 9 amino acids, all of which maintaining the conserved FxxGFG motif (Fig. 4*G*). We next tested the capacity of each peptide to inhibit the reconstitution of the uS5-PDCD2 biosensor. As shown in Figure 4*H*, all shorter versions of uS5-derived peptides, except for the 9 amino acid long uS5_24-32_ peptide, inhibited the reconstitution of the uS5-PDCD2 biosensor as effectively as the 30 amino acid-long wild-type uS5_21-50_ peptide. The establishment of a robust biosensor that quantitatively measures uS5-PDCD2 interaction *in cellulo* and *in vitro*, allowed the identification of a short uS5-based peptide, corresponding to residues 22 to 32, which can effectively inhibit uS5-PDCD2 interaction.

### uS5-derived peptides impair viability in cancer cell lines

**Figure 5:**
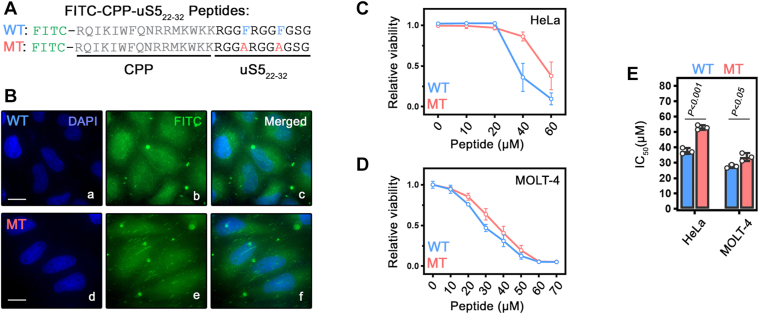
**uS5-derived peptides inhibit cancer cell growth.***A*, Amino acid sequences of wild-type (WT) and F25A/F29A mutant (MT) CPP-uS5 (22–32) peptides, with an N-terminal FITC fluorophore. *B*, fluorescence microscopy images of HeLa cells incubated with 20 μM WT or MT CPP-uS5 (22–32) peptides. Cells were fixed 24 h post-incubation and analyzed by direct FITC fluorescence (panels b and e). Nuclei were counterstained with DAPI (panels a and d). Scale bars, 20 μm. *C-D*, Dose–response analysis of cell viability in HeLa (*C*) and MOLT-4 (*D*) cells following 24 h of peptide treatment. Data represent mean ± SD from three independent experiments. *E*, Half-maximal inhibitory concentration (IC_50_) values were determined by fitting dose-response curves using nonlinear regression analysis. Statistical differences were calculated using student t-tests. *p*-values are indicated.

To test whether selective disruption of the PDCD2–uS5 interaction by synthetic uS5-derived peptides could be translated into inhibition of cancer cell viability, we generated cell-penetrating versions of both the wild-type (WT) and mutant (MT; F25A/F29A) uS5_22–32_ peptides by adding the Antennapedia (Penetratin) cell-penetrating peptide (CPP) sequence (Fig. 5*A*). Penetratin, a 16-amino-acid sequence derived from the *Drosophila* Antennapedia homeodomain, is a well-established CPP that has been widely used to deliver diverse cargoes into cells (33). To verify intracellular uptake, we covalently attached a fluorescein isothiocyanate (FITC) label to the N-terminus of both peptides (Fig. 5*A*). Fluorescence microscopy confirmed internalization of both wild-type and mutant CPP-uS5-derived peptides in HeLa cells (Fig. 5*B*).

We next assessed the impact of these peptides on cell viability using resazurin-based assays in HeLa cells across increasing peptide concentrations. As shown in Figure 5*C*, the wild-type CPP-uS5-derived peptide induced a dose-dependent reduction in cell viability over 24 h (blue curve), with an IC_50_ of approximately 36 μM (Fig. 5*E*). In contrast, the mutant CPP-uS5-derived peptide, designed to weaken competition for the uS5-PDCD2 interaction, showed a reduced growth-inhibitory effect (Fig. 5*C*, red curve), with an IC_50_ of about 55 μM (Fig. 5*E*). Because PDCD2 has been implicated in hematopoiesis (34), we also examined the effects of the uS5_22-32_ peptides in MOLT-4 cells, a hematopoietic T-lymphoid leukemia suspension cell line. Although overall growth inhibition was less pronounced in MOLT-4 than in HeLa cells (Fig. 5*D*), the wild-type peptide still decreased cell viability more strongly than the mutant peptide (Fig. 5*E*). Taken together with our binding data, these results support the conclusion that interfering with the uS5-PDCD2 interaction impairs cancer cell proliferation.

## Discussion

In this study, we define the molecular basis by which the human 40S ribosomal protein uS5 is specifically recognized by its dedicated chaperone PDCD2. We identify a minimal PDCD2-interacting region within the eukaryote-specific N-terminal extension of uS5 (amino acids 21–50) and demonstrate that conserved phenylalanine residues within this region are critical determinants of molecular recognition. Structural modeling and mutational analyses support a mechanism in which F25 and F29 insert into hydrophobic pockets on PDCD2 to stabilize complex formation (Fig. 2). In full-length uS5, these residues contribute additively to PDCD2 binding, whereas in the isolated minimal domain each phenylalanine is essential, indicating that additional contacts reinforce complex stability in the native context. Together, our data support a model in which F25 and F29 function as anchor residues that promote co-translational capture of nascent uS5 by PDCD2.

Recognition of N-terminal elements appears to be a conserved feature of ribosomal protein-dedicated chaperone systems. Consistent with our findings, the yeast PDCD2 homolog Tsr4 binds the extreme N-terminus of uS5 (18, 21), and several other dedicated chaperones similarly engage N-terminal regions of their ribosomal protein clients (17, 19, 35, 36). Such positioning would enable early capture of nascent polypeptides during translation, limiting aggregation or degradation. More broadly, insertion of phenylalanine side chains into hydrophobic clefts represents a well-established mode of protein-protein interaction (37, 38). The conservation of the uS5 FxxGFG motif (Fig. S1) and structural predictions in yeast uS5 showing phenylalanine insertion into Tsr4 (Fig. S6), suggest that hydrophobic anchoring of conserved phenyl residues constitutes a shared evolutionary strategy for uS5 recognition.

Beyond defining the interaction interface, we investigated functional consequences of the uS5 N-terminal region. The Glycine-Arginine-rich (GAR) domain encompassing residues 21 to 50 was sufficient to target GFP to nucleoli (Figs. 3 and S3), consistent with the known nucleolar targeting properties of GAR domains (39, 40, 41), potentially by facilitating biophysical behavior such as phase separation (42). Notably, a mutant GAR domain defective in PDCD2 binding retained nucleolar enrichment, indicating that nucleolar localization of this region is largely independent of PDCD2. In *S. cerevisiae*, uS5 accumulates in the nucleus of cells deficient for Tsr4 (21, 43), supporting that Tsr4 is not required for nuclear import of uS5 in yeast. Intriguingly, excess expression of the minimal uS5 domain altered the subcellular distribution of PDCD2, leading to increased cytoplasmic accumulation (Fig. 3, *C* and *D*). These observations, together with data from bimolecular fluorescence complementation (BiFC) assays (24), are consistent with a model in which PDCD2 associates with uS5 prior to nuclear import; yet, whether PDCD2 nuclear entry strictly depends on uS5 binding remains to be determined.

A major advance of this work is the development of a robust and sensitive NanoBiT-based complementation biosensor to monitor uS5-PDCD2 interactions in living human cells. Unlike endpoint assays or irreversible BiFC approaches, this system enables quantitative and reversible detection of protein-protein interactions in real time. Optimization of tag orientation revealed that N-terminal fusions of both partners produced the strongest signal, consistent with structural predictions placing their N-termini in close proximity within the complex (23). In fact, the model of the uS5-PDCD2 complex predicts that the C-terminal end of PDCD2 is concealed, which is consistent with our results showing that both PDCD2-LgBiT and PDCD2-SmBiT fusions poorly reconstituted a functional NanoBiT luciferase (Fig. 4). This biosensor therefore provides a physiologically relevant platform to interrogate the dynamics and regulation of ribosomal protein-chaperone interactions.

Finally, leveraging this system, we identified short uS5-derived peptides that competitively disrupt uS5-PDCD2 binding in cells and *in vitro*. Disruption was dose-dependent and required the conserved phenylalanine residues, demonstrating specificity and validating the predicted binding interface. Given that PDCD2 is broadly required for cancer cell fitness according to the DepMap project (28), targeting ribosomal protein-dedicated chaperone interactions may represent a novel therapeutic strategy to selectively impair ribosome biogenesis and cancer cell viability, as supported by our data (Fig. 5). Thus, our study not only elucidates the structural logic of PDCD2-mediated uS5 recognition but also establishes a versatile platform for high-throughput screening of compounds that disrupt uS5-PDCD2 binding. By coupling fundamental discovery with a powerful screening tool, our work lays the foundation for exploring ribosomal protein dedicated chaperones as a novel and selective therapeutic vulnerability in cancer.

## Experimental procedures

### Cell culture

Human MOLT-4, HEK293T, HeLa, and U2OS cell lines were acquired from ATCC. Cell lines were grown in Dulbecco’s Modified Eagle Medium (DMEM) (Wisent # 319-005 CL), supplemented with 10% Fetal Bovine Serum (Corning #35-077-CV), 1% Penicillin-Streptomycin (Wisent # 450-202 EL), and 1% nonessential amino acids (Wisent #321-011 EL) at 37 °C in 5% CO_2._ MOLT-4 was grown Roswell Park Memorial Institute (RPMI1640) (Wisent #350-200-CL) medium supplemented with 10% Fetal Bovine Serum (Corning #35-077-CV), 1% Penicillin-Streptomycin (Wisent # 450-202 EL), and 1% nonessential amino acids (Wisent #321-011 EL) at 37 °C in 5% CO2. U2OS-Flag-PDCD2 and HeLa-uS5-YFP stable conditional cell lines were previously described (24) and induction of transgenes was induced using doxycycline at a final concentration of 1000 ng/ml. Lipofectamine 2000 (Invitrogen #11668019) was used for all transfections (except for NanoBiT biosensor constructs) according to manufacturer’s instructions. siRNAs were transfected at a final concentration of 20 nM. Transfections of NanoBiT constructs were done using PolyJet reagent (SignaGen # SL100688) according to the manufacturer’s instruction. Briefly, plasmid DNA and PolyJet were diluted in serum-free DMEM in a 1:3 ratio (3ul PolyJet: 1ug DNA), mixed, and incubated at room temperature for 15 min. The transfection mix was then added on top of each culture.

### Plasmid constructs and mutagenesis

uS5-YFP plasmid has been described previously (24). To create truncated versions of uS5, the uS5-YFP construct was used in a PCR reaction using primers designed to flank regions of interest. PCR products were then purified and cloned into pEGFP-N1 under a CMV promoter and in frame with a C-terminal GFP tag using a Gibson Assembly kit (NEB #E2621G). For site-directed mutagenesis, primers containing the desired mutations, either at the 5′ end or in the middle of the primer, were designed. Wild-type plasmids were used as templates in PCR reactions. PCR products were purified and then subjected to either KLD (NEB #M0554) or Quick Change. Flag-PDCD2 and PDCD2-GFP plasmids were described previously (24). All constructs were sequenced using Sanger sequencing at the Sanger Sequencing Platform (Université Laval). Due to the large number of primers used in this study, sequences are available upon request.

### Protein analyses and antibodies

For protein extraction, cells from 6-well plate format were harvested in 1 ml ice cold PBS and pelleted by centrifugation for 3 min at 1500 rpm at 4 °C. Pellets were resuspended in 100 μl lysis buffer (50 mM Tris-HCl pH 7.5, 150 mM NaCl, 0.1% Triton X-100, 10% glycerol, 2 mM MgCl2) supplemented with Complete protease inhibitor cocktail (Roche #11873580001) and incubated for 15 min on ice. Lysates were cleared by centrifugation at 13,000 rpm for 10 min at 4 °C, and the supernatant was kept for protein analyses. For coimmunoprecipitation experiments, ∼90ul lysates were subjected to 25 μl of GFP-trap beads (ProteinTech) or anti-FLAG M2 affinity gel (Millipore, # A2220). Briefly, beads were pre-washed three times for 1 min each with the 1 ml lysis buffer and resuspended in lysis buffer supplemented with protease inhibitor cocktail. Lysates were incubated with the beads for 2 h at 4 °C with constant rotation, beads were pelleted and washed 5 times for 1 min with 1 ml of lysis buffer supplemented with protease inhibitor cocktail. Proteins were eluted from the beads in 25 μl 1X SDS-loading buffer (63 mM Tris-HCl pH 6.8, 10% glycerol, 2% sodium dodecyl sulfate, 0.1 M DTT, bromophenol blue). For Western blot analyses, proteins were separated by SDS-PAGE and transferred onto nitrocellulose membranes. Membranes were blocked with 5% skim-milk and then probed with primary antibodies against PDCD2 (Abcam, #ab133324), uS5 (Santa Cruz Biotechnology, #sc-130399), Tubulin (Sigma, #T5168), Actb (Sigma, #A5441), GFP (Roche, #11814460001), and Flag (Sigma, #F1804). Secondary donkey anti-rabbit antibody conjugated to IRDye 800CW (Li-Cor, #926-32213) and goat anti-mouse antibody conjugated to Alexa Fluor 680 (Life Technologies, #A-21057) were used to visualize bands on an Odyssey Classic (LI-COR). Band intensities were quantified with the Odyssey Software V3.0 (LI-COR).

### Fluorescence and confocal microscopy

For PDCD2 localization, U2OS-FT cells that conditionally express Flag-PDCD2 were grown on top of coverslips in a 6-well plate and transfected with either wild-type or mutant version of uS5_21-50_-GFP. Flag-PDCD2 expression was then induced using doxycycline for 24 h. For uS5_21-50_-GFP localization, U2OS-FT cells were transfected with either wild-type or mutant uS5_21-50_-GFP for 24 h, as described for PDCD2. For Peptide uptake visualization, HeLa cells were grown on top of a coverslips in a 6-well plate in presence of either wild-type or mutant version of the FITC-tagged peptides. Coverslips were fixed in paraformaldehyde (4%) for 15 min at room temperature and permeabilized with ice-cold ethanol (75%) for 10 min, which maintained detectable native GFP fluorescence. DAPI (Sigma, #D9542) and antibody staining against Fibrillarin (Cell Signalling, #2639) were used to stain nucleus and nucleolus, respectively. Flag-PDCD2 was stained using anti-Flag (Sigma, #F1804). Both Flag and Fibrillarin were visualized using Alexa Fluor 568–conjugated secondary antibody (Invitrogen #A11019 and #A21069, respectively). Microscopy analysis was done on a Zeiss LSM880 or a Zeiss LSM700. Representative micrographs were taken in stacks of 8 z-section, on a 1.5 μm intervals. Sections were then deconvoluted using ZEN2.6 software and stacked using a customized plugin in ImageJ (44). CellProfiler 4.1.3 software (45) was used to measure nuclear-to-cytoplasmic and nucleolar-to-nuclear ratios, as previously described (46).

### uS5-PDCD2 biosensor assay

Plasmids designed for the NanoBit-based biosensor assay were synthesized by GenScript. For the development of the NanoBiT-based uS5-PDCD2 interaction biosensor, the coding sequence of *uS5* and *PDCD2* genes was codon-optimized and cloned under a CMV promoter into pcDNA3.1(+) in frame with a 3xFlag tag at the N-terminus, and either SmBiT or LgBiT protein sequences fused at the N- or C-terminus, as well as a 10xHis tag fused at the C-terminus. Considering the position and combination of the tags, a total of 8 plasmids (4 with uS5 and 4 with PDCD2) were designed. To select the best uS5-PDCD2 combination, plasmids were cotransfected into HEK293T cells. 36 h post-transfection, cells were rinsed with ice-cold PBS and resuspended in lysis buffer (Invitrogen, # FNN0021). After 10′ incubation on ice, lysates were transferred to a 1.5-ml tube and lysates were cleared by centrifugation at 13000 rpm at 4 °C. Supernatants were collected and 50-ul of each lysate was loaded into a 96-well opaque plate for bioluminescence reading. Coelenterazine (CTZ, NanoLight, #303) was added to each well at a final concentration of 3.6 uM. For assays in living human cells, 36 h post-transfection, cells grown in 96-well format were incubated in fresh media supplemented with CTZ for 30 min, washed with warm PBS once, and supplemented with 50 ul of warm PBS containing CTZ. Bioluminescence emission was read on a Biotek Synergy plate reader at 500 msec and gain set at 220.

### Solid-phase peptide synthesis

The uS5-derived peptides were synthesized on a Symphony X Peptide Synthesizer (Gyros Protein Technologies) using standard Fmoc solid-phase peptide synthesis, as described previously (47). Briefly, the synthesis was performed on a 0.04 mmol scale employing TentaGel S RAM resin (loading: 0.23 mmol/g). Coupling reactions were carried out with 5 equiv of Fmoc-protected amino acid, 5 equiv of HATU, and 20 equiv of *N*-methylmorpholine in DMF. Fmoc deprotection was performed using 20% piperidine in DMF. After peptide chain elongation, the peptides were cleaved from the resin and the side chain protecting groups were simultaneously removed using a trifluoroacetic acid/H_2_O/triisopropylsilane (95:2.5:2.5, v/v/v) cocktail for 2 h at room temperature. The peptides were purified by Waters preparative HPLC-MS (column XSELECTTM CSHTM Prep C18 (19 × 100 mm) packed with 5 μm particles, UV detector 2998, MS SQ Detector 2) using a gradient of 5% to 95% acetonitrile (0.1% FA) in water (0.1% FA). All synthetic peptides were verified by UPLC-MS to confirm identity and purity (>95%).

### Cell viability assays

To assess the effect of PDCD2-uS5 interaction inhibition using the peptides on cell growth, we used Sigma’s Resazurin-based In Vitro Toxicology Assay Kit (Sigma, #TOX8-1KT). Viability tests were performed according to manufacturer’s instructions. Briefly, HeLa and MOLT-4 cells were seeded in a 96-well format at sub-confluent densities and incubated overnight for adaptation. The day after, peptides were added to the media and cells were incubated for 24 h. To measure the viability, Resazurin was added at a final concentration of 10% (v/v) followed by a 2-h incubation time. Fluorescent signal was measured at 590 nm using a Molecular Devices Flex Station 3 plate reader at 590 nm (excitation at 560 nm), at 37 °C. Viability values are reported relative to no treatment (0) controls. IC_50_ values were calculated by GraphPad Prism 6.

## Data availability

All data will be shared upon requests to the corresponding author.

## Supporting information

This article contains Supporting Information, including unprocessed original images of Western blots.

## Conflict of interest

The authors declare that they have no conflicts of interest with the contents of this article.
