# Supplementary material for: Biosensor-guided discovery of peptide inhibitors targeting the ribosomal protein uS5-PDCD2 chaperone interaction
Source: J Biol Chem. 2026 Mar 31;302(5):111415. doi: 10.1016/j.jbc.2026.111415 (PMC13127195; doi:10.1016/j.jbc.2026.111415)

SUPPORTING INFORMATION

Biosensor-Guided Discovery of Peptide Inhibitors Targeting the Ribosomal Protein uS5–PDCD2 Chaperone Interaction

Zabih Mir Hassani, Frédérique Goulet, Duc Tai Nguyen, Anne-Marie Landry-Voyer, Lauren Kwiatek, Shany Gaudet, Pierre-Luc Boudreault, Taha Azad, François Bachand.

Inventory:

- 6 supplementary figures
- Unprocessed original images of western blot results

SUPPLEMENTARY FIGURES

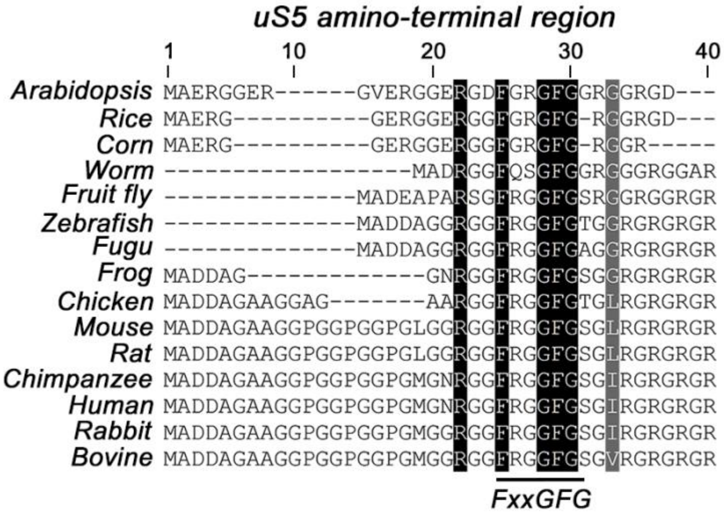

Supplementary Figure 1. Evolutionary conservation of the FxxGFG motif in the N-terminal region of uS5. Multiple sequence alignment of the N-terminal region of uS5 from the indicated species. *Top*, numbering of amino acids 1-40 is for human uS5. The alignment was generated using ClustalW and visualized with Boxshade. The conserved FxxGFG motif is underlined.

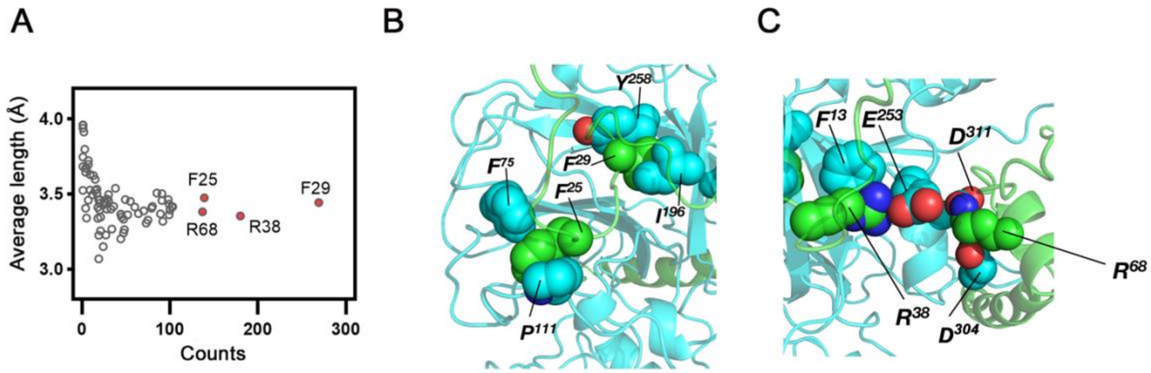

Supplementary Figure 2. ***Atomic contact analysis of human uS5 predicted by AlphaFold.*** **(A)** Each point represents a residue from human uS5, based on AlphaFold-predicted atomic coordinates. The x-axis indicates the number of atomic contacts formed by each amino acid with PDCD2, while the y-axis shows the average interatomic distance (Å) for all contacts within a 4 Å cutoff. Contacts were computed by measuring pairwise atomic distances between all atoms of the uS5-PDCD2 pair and retaining those below the threshold. Residues indicated in red are expected to exhibit a high number of short-distance contacts and would correspond to potential interface "hot spots", suggesting regions of strong intermolecular interaction and structural complementarity. **(B)** AlphaFold-predicted complex highlighting the hydrophobic sandwich arrangement in which F25 and F29 of uS5 (shown in green) are intercalated between aromatic residues (F75 and P111 for F25; I196 and Y258 for F29) of PDCD2 (shown in cyan). **(C)** R38 and R68 of uS5 (green) form salt bridge interactions with E253 and D304/D311, respectively, of PDCD2 (cyan). Blue, positively charged arginines forming salt bridges. Red, negatively charged aspartate/glutamate residues forming complementary electrostatic partners.

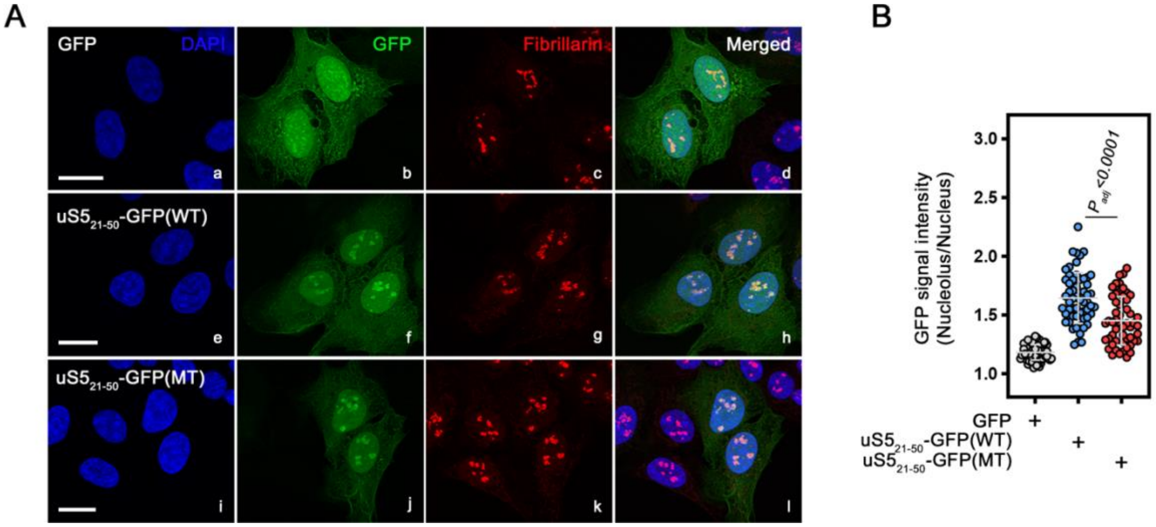

Supplementary Figure 3. ***uS5$_{21-50}$-GFP is targeted to the nucleolus independently of PDCD2*****. (A)** HeLa cells were transfected with either GFP control vector (panels a-d), wild-type uS5$_{21-50}$-GFP (WT, panels e-h), or the F29Y mutant version of uS5$_{21-50}$-GFP (MT, panels i-l). 36h post-transfection, cells were fixed and simultaneously analysed by direct fluorescence (b, f and j) and immunostaining for Fibrillarin (c, g and k). DNA staining with DAPI shows the nucleus of each cell (a, e and i). Scale bars, 20 µm. **(B)** Quantification of nucleolus-to-nucleus ratios of GFP signal. More than 60 cells were analyzed for each condition, with at least two independent immunofluorescence experiments. Statistical differences were calculated using a one-way ANOVA with Dunnett's multiple comparisons test. P-values are indicated.

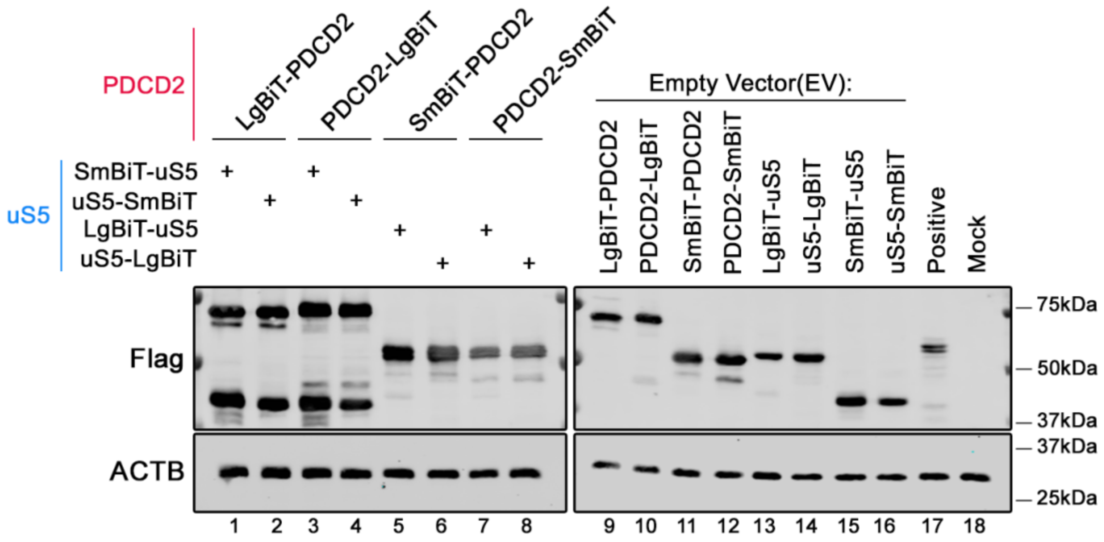

Supplementary Figure 4. ***Expression of LgBiT and SmBiT fusion proteins used for the NanoBiT complementation-based biosensor***. Western blot analysis of total extracts (lanes 1-18) prepared from HEK293T cells that were transiently transfected for 48h with the indicated constructs. Note that SmBiT fusions with PDCD2 (lanes 5-8 and 11-12) and LgBiT fusions with uS5 (lanes 5-8 and 13-14) have similar molecular weights and comigrate on SDS-PAGE (lanes 11-14), resulting in a single band when co-transfected (lanes 5-8). The blot was analyzed for Flag (top) and beta-actin (bottom)

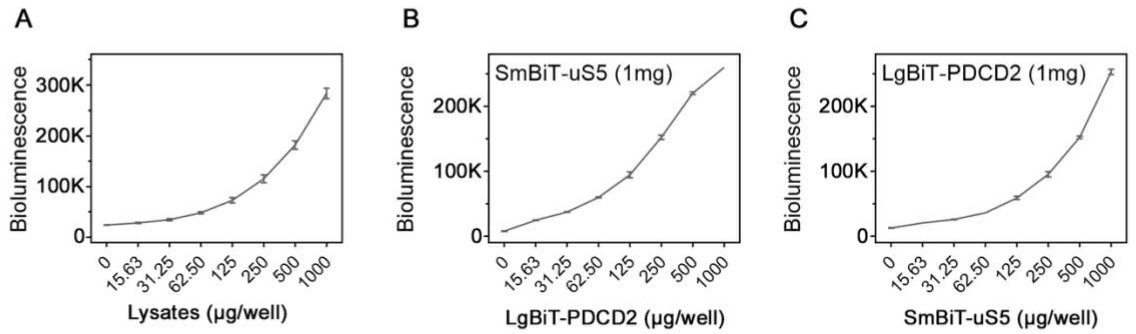

Supplementary Figure 5. ***Dose-dependent reconstitution of NanoBiT luciferase by mixing extracts expressing LgBiT-PDCD2 and SmBiT-uS5.*** HEK293T cells were transfected with plasmid constructs expressing either LgBiT-PDCD2 or SmBiT-uS5 for 48h, lysed, quantified, and (A) equal amounts of lysates were mixed at increasing doses. Mixed lysates were incubated at 25°C for 15 minutes, coelenterazine (CTZ) was added, and the bioluminescence signal was read on a luminometer. In (B), increasing doses of LgBiT-PDCD2 lysate were added in each well. Then, 1 mg of SmBiT-uS5 lysate was added, and the mixed lysates were incubated at 25°C for 15 minutes. Coelenterazine (CTZ) was added, and the bioluminescence signal was read on a luminometer. In (C), increasing doses of SmBiT-uS5 lysate were added in each well. Then, 1 mg of LgBiT-PDCD2lysate was added, and the mixed lysates were incubated at 25°C for 15 minutes. Coelenterazine (CTZ) was added, and the bioluminescence signal was read on a luminometer. All experiments were performed in triplicate, and error bars represent the standard deviation of the mean.

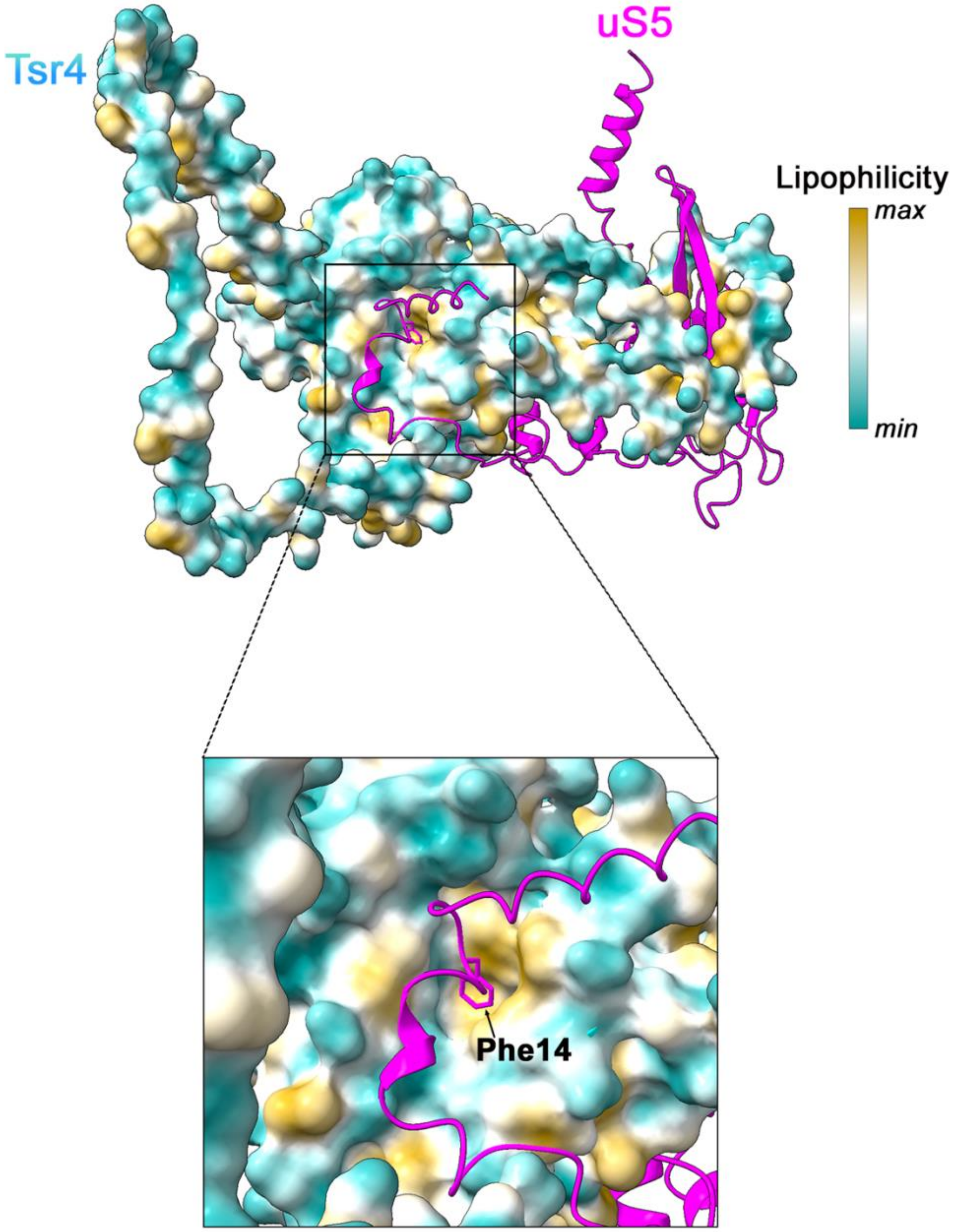

Supplementary Figure 6. Phe14 of *S. cerevisiae* uS5 is buried inside a hydrophobic pocket region in Tsr4. (*Top*) AlphaFold 3-predicted model of the *S. cerevisiae* uS5-Tsr4 complex. The ribbon-like structure of uS5 is shown (Magenta) together with the surface representation of Tsr4 lipophilicity, with yellow and blue showing the maximum and minimum lipophilicity, respectively. (*Bottom*) A zoom on a region of the uS5-Tsr4 complex showing that Phe14 of *S. cerevisiae* uS5 is predicted to be embedded in a hydrophobic core region of Tsr4.

Uncropped raw images of western blots presented in this study

- Below are the uncropped raw Western blot images exported from the Li-Cor system.
- Molecular weight markers (kDa) are indicated on the left side of the ladder.
- Red boxes denote the regions cropped and presented in the manuscript figures.
- Red arrows indicate the orientation of the blot (left-to-right or right-to-left) as shown in the figures.

Figure 1B

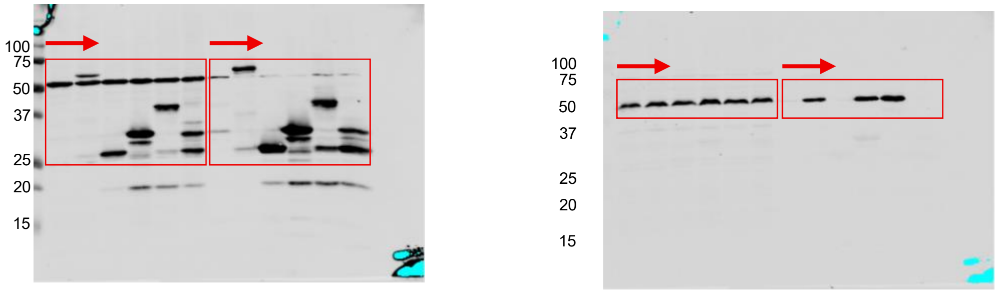

Figure 1C

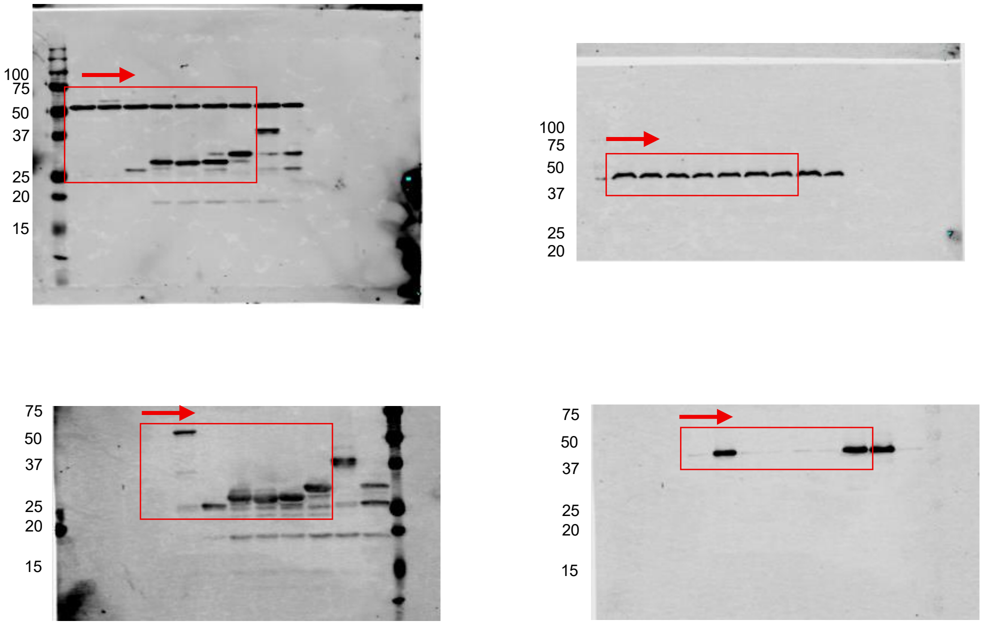

Figure 1D

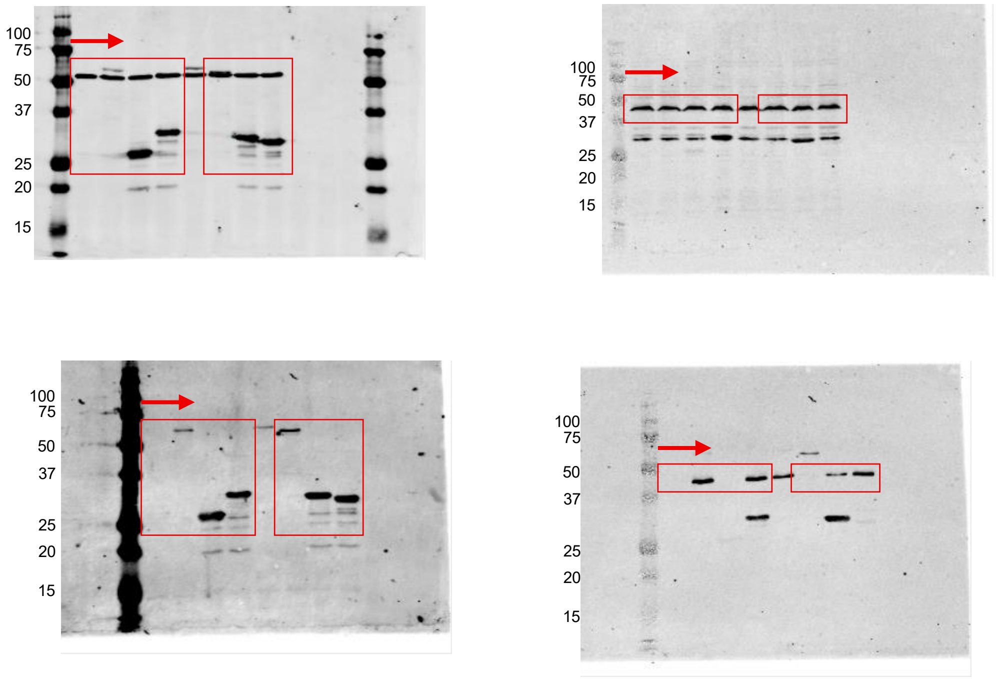

Figure 2D

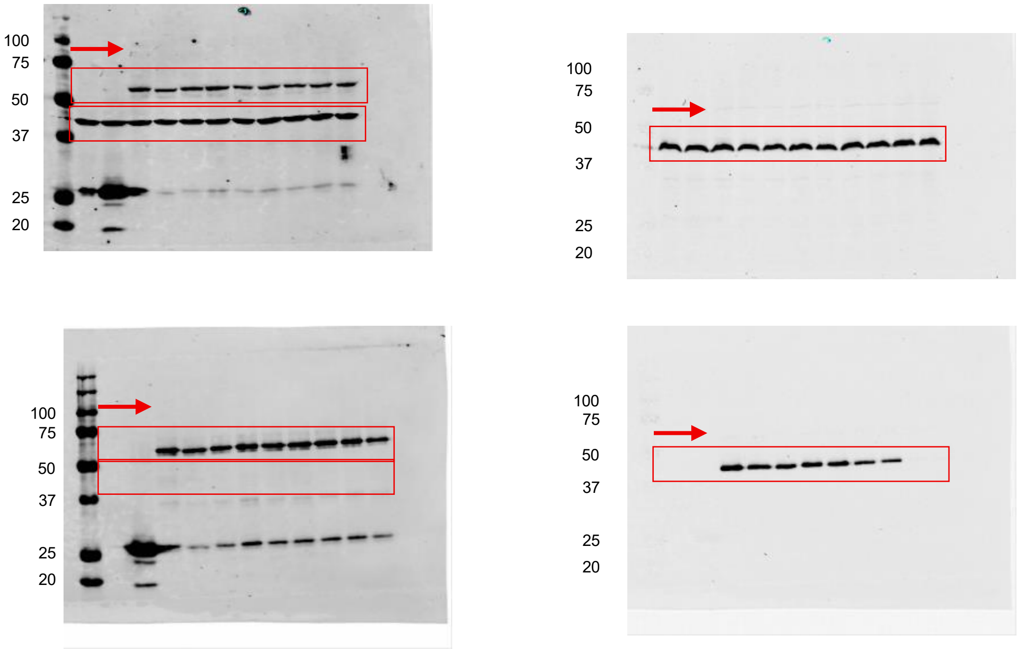

Figure 2F

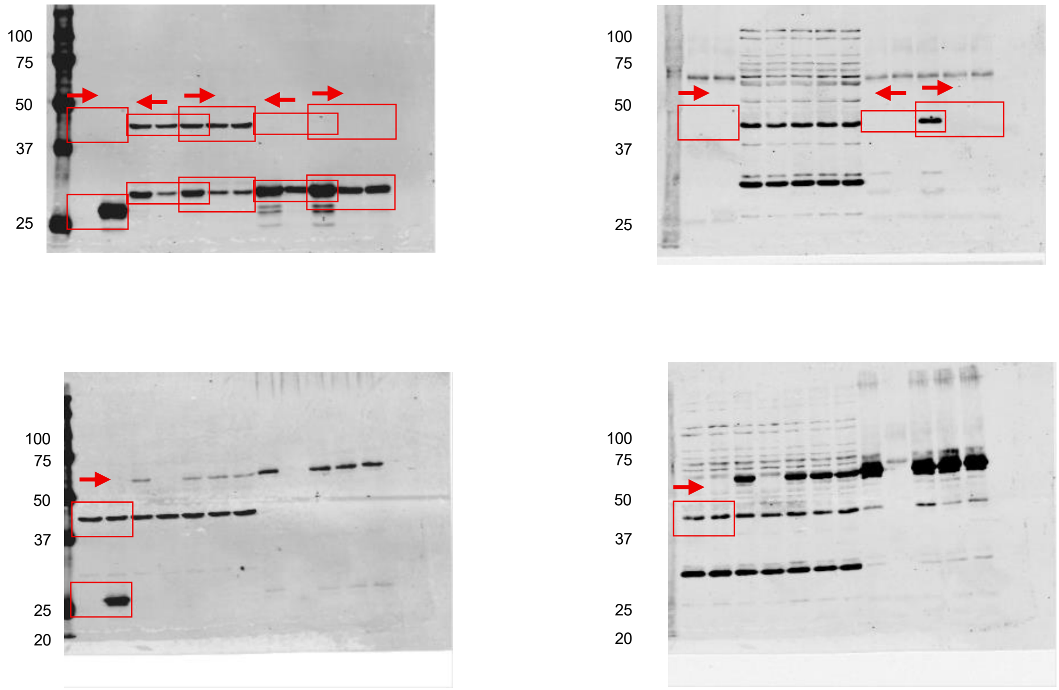

Figure 2I

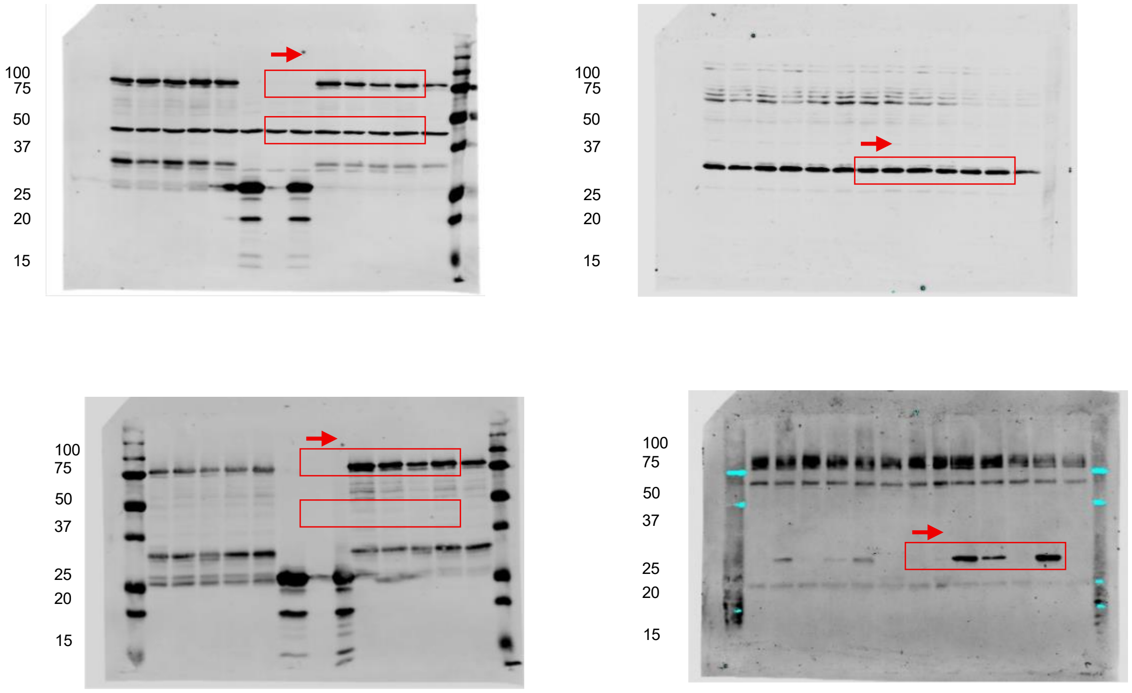

Figure 3B

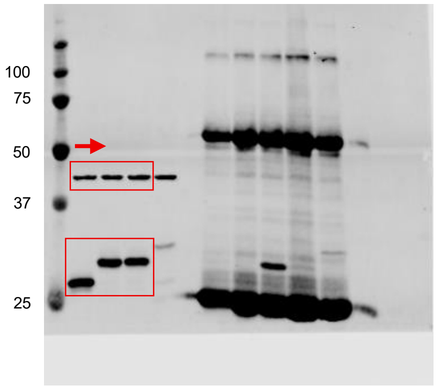

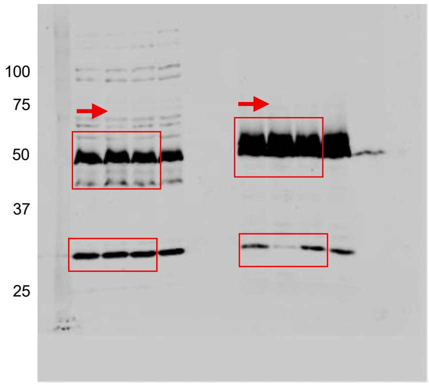

Figure 3G

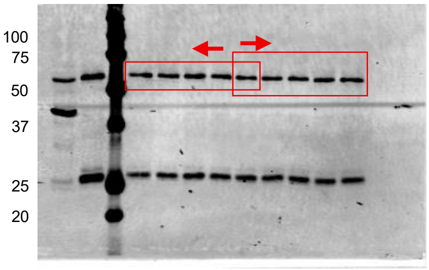

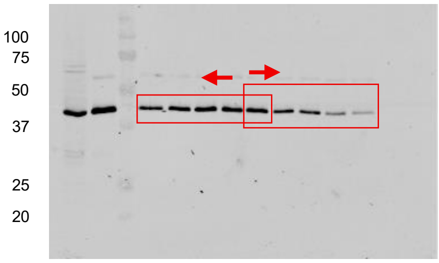

Supplement: Supporting Figures [file mmc1.pdf]
